# Induction of p53-independent growth inhibition in lung carcinoma cell A549 by gypenosides

**DOI:** 10.1111/jcmm.12546

**Published:** 2015-03-17

**Authors:** Jung-Sen Liu, Tzu-Hsuan Chiang, Jinn-Shyan Wang, Li-Ju Lin, Wei-Chih Chao, Baskaran Stephen Inbaraj, Jyh-Feng Lu, Bing-Huei Chen

**Affiliations:** a School of Medicine, Fu Jen Catholic UniversityNew Taipei City, Taiwan; b Department of Surgery, Cathay General HospitalTaipei, Taiwan; c Graduate Institute of Medicine, Fu Jen Catholic UniversityNew Taipei City, Taiwan; d Department of Food Science, Fu Jen Catholic UniversityNew Taipei City, Taiwan

**Keywords:** *Gynostemma pentaphyllum*, saponin, carotenoid, chlorophyll, lung cancer cell

## Abstract

The objectives of this study are to investigate antiproliferative effect and mechanisms of bioactive compounds from *Gynostemma pentaphyllum* (*G. pentaphyllum*) on lung carcinoma cell A549. Saponins, carotenoids and chlorophylls were extracted and fractionated by column chromatography, and were subjected to high-performance liquid chromatography-mass spectrometry analyses. The saponin fraction, which consisted mainly of gypenoside (Gyp) XXII and XXIII, rather than the carotenoid and chlorophyll ones, was effective in inhibiting A549 cell growth in a concentration- and a time-dependent manner as evaluated using 3-(4,5-dimethylthiazol-2-yl)-2,5-diphenyltetrazolium bromide (MTT) assay. The estimated half maximal inhibitory concentration (IC_50_) of Gyp on A549 cells was 30.6 μg/ml. Gyp was further demonstrated to induce an apparent arrest of the A549 cell cycle at both the S phase and the G2/M phase, accompanied by a concentration- and a time-dependent increase in the proportions of both the early and late apoptotic cells. Furthermore, Gyp down-regulated cellular expression of cyclin A and B as well as BCL-2, while up-regulated the expression of BAX, DNA degradation factor 35 KD, poly [ADP-ribose] polymerase 1, p53, p21 and caspase-3. Nevertheless, both the treatment of a p53 inhibitor, pifithrin-α, and the small hairpin RNA-mediated p53 knockdown in the A549 cells did not alter the growth inhibition effect induced by Gyp. As a result, the cell cycle arrest and apoptosis of A549 cells induced by Gyp would most likely proceed through p53-independent pathway(s).

## Introduction

*Gynostemma pentaphyllum* (*G. pentaphyllum*), commonly known as ‘Cheap Ginseng’, is widely distributed and consumed in Asian countries, especially Taiwan, China, Japan and Korea, with several vital biological activities, including anti-cancer, anti-inflammation and anti-atherosclerosis, reported to be associated with the intake of *G. pentaphyllum* 1,2. The presence of various bioactive components, such as saponins, carotenoids and chlorophylls, is believed to play a pivotal role in the prevention of chronic diseases. The saponins from *G. pentaphyllum* contain mainly dammarane triterpene saponin glycosides commonly referred to as gypenosides (Gyp), several of which are identical to those found in ginseng (*Panax*). The effect of Gyp on anti-cancer activity has been well-documented. For examples, Gyp were shown to exhibit antiproliferative effect on hepatoma cells Hep3B 2 and Huh7 3, prostate cancer cell PC-3 4, tongue cancer cell SCC4 5 and leukaemia cell WEHI-3 6 *via* cell cycle arrest and/or apoptosis. However, many of the details of molecular mechanisms underlying this anti-cancer activity still await to be unveiled and confirmed.

Both carotenoids and chlorophylls are widely present in plants, especially green vegetables. Carotenoids belong to a group of lipid-soluble compounds with colour ranging from yellow to red and are divided into carotene and xanthophyll with the former containing only hydrocarbon and the latter containing oxygen derivatives 7. Numerous reports have been published over the protective effect of carotenoids against cancers. For instance, lycopene, a well-known carotenoid possessing strong antioxidative activity, is a potential agent for prevention and treatment of prostate cancer through induction of G0/G1 cell cycle arrest and suppression of phosphatidylinositol 3-kinase-dependent proliferative and survival signalling in androgen-responsive LNCaP and androgen-independent PC-3 cells 8. Likewise, β-carotene, a major carotenoid in green plants and fruits, is capable of inducing differentiation and apoptosis of leukaemia cells HL-60 and U937 9.

Like carotenoids, chlorophylls are also the most abundant pigments in green plants and usually constitute a larger portion than carotenoids. The dominant chlorophylls in green plants include chlorophyll a and chlorophyll b, which are present at an approximate ratio of 3:1. Several chlorophyll derivatives, including chlorophyllide, pheophorbide and chlorophyllin, have been shown to be cancer-preventive through enhancement of antioxidant and antimutagenic activity, modulation of xenobiotic metabolism, and induction of apoptosis 10.

Worldwide, lung cancer is the most common human malignancy in terms of both incidence and mortality. From the 1960s, the rates of lung carcinoma started and continued to rise compared to other types of lung cancer. Although potential growth inhibitory effect of Gyp, triterpenoid saponins extracted from *G. pentaphyllum*, on human lung cancer cells has been implicated 11, the effect of other bioactive compounds and the underlying molecular mechanism still remain to be confirmed. The objectives of this study are to investigate and elucidate potential antiproliferative effect and relevant mechanisms of saponins, carotenoids and chlorophylls from *G. pentaphyllum* on lung carcinoma A549 cell.

## Materials and methods

### Chemicals

*Gynostemma pentaphyllum* was purchased from a local herbal dealer in Taipei, Taiwan. Carotenoid standards, including all-trans-lutein and all-trans-β-apo-8′-carotenal (internal standard), were obtained from Fluka Chemical Co. (Buchs, Switzerland). All-trans-β-cryptoxanthin was from Extrasynthese Co. (Genay, France), while all-trans-α-carotene and all-trans-β-carotene were from Sigma-Aldrich (St. Louis, MO, USA). Both chlorophyll a and chlorophyll b standards were also from Sigma-Aldrich. Internal standard Fast Green FCF was from Fluka Chemical Co. Both pheophytin a and pheophytin b standards were prepared by dissolving 1 mg of chlorophyll a and chlorophyll b in 1 ml of acetone, respectively, followed by adding 2–3 drops of 0.1 N methanolic hydrogen chloride solution, shaking, evaporating to dryness under nitrogen and dissolving in 1 ml of acetone.

The high-performance liquid chromatography (HPLC)-grade solvents, including methanol, acetonitrile, methylene chloride and acetone, were procured from Lab-Scan Co. (Gliwice, Poland). The analytical-grade solvents, including hexane, toluene, ethanol, acetone and ethyl acetate, were from Lab-Scan and Grand Chemical (Taipei, Taiwan). Deionized water was obtained by a Milli-Q water purification system from Millipore Co. (Bedford, MA, USA). Adsorbents such as magnesium oxide and diatomaceous earth were from Sigma-Aldrich and J. T. Baker (Phillipsburg, NJ, USA) respectively.

### Reagents

Cell culture reagents, including F-12K medium, foetal bovine serum (FBS), penicillin-streptomycin and 2.5% trypsin-EDTA, were from Gibco Life Technologies (Grand Island, NY, USA). Trypan blue stain 0.4% solution was from Invitrogen (Carlsbad, CA, USA). Anti-β-actin and MTT reagent 3-(4,5-dimethylthiazol-2-yl)-2,5-diphenyltetrazolium bromide were from Sigma-Aldrich. Ribonuclease A (RNase A) was from Roche (Basel, Switzerland). SDS, dimethyl sulphoxide (DMSO), Tween 20, 40% Acrylamide/Bis and TEMED were from J. T. Baker. Propidium iodide (PI) and Annexin-V were from BD Biosciences Co. (San Diego, CA, USA). Pifithrin-α (PFTα) was from BioVison, Inc. (San Francisco, CA, USA). The primary antibodies, including anti-cyclin A and B, anti-DNA degradation factor 45 KD (DFF45), anti-p21 and anti-cytochrome c, were from BD Biosciences Co. (San Jose, CA, USA), while anti-caspase-3, anti-BCL-2, anti-BCL-xL, anti-BAD, anti-BAX, anti-poly [ADP-ribose] polymerase 1 (PARP-1) and anti-p53 were from Epitomics Co. (Burlingame, CA, USA). Anti-cyclin E was from Thermo Fisher Scientific (Waltham, MA, USA). The secondary antibodies, such as goat anti-rabbit IgG-horseradish peroxidase (HRP) and rabbit anti-mouse IgG-HRP, were from Chemicon Co. (Temecula, CA, USA). Human lung carcinoma cell line A549 was from Bioresource Collection and Research Center, Taiwan Food Industry Development and Research Institute/National Research Institute of Health (Hsinchu, Taiwan). Small hairpin RNA (shRNA) reagents were obtained from the National RNAi Core Facility at the Institute of Molecular Biology/Genomic Research Center, Academia Sinica (Taipei, Taiwan).

### Instrumentation

The Agilent 1100 Series HPLC system was composed of a G1311A pump, a G1312A pump, a G1316A column temperature controller, a G1379A on-line degasser, a G1315B photodiode-array detector, and a 6130 quadrupole mass spectrometer with multi-mode ion source (EI and APCI). The flow cytometer was from Partec (Münster, Germany). The inverted microscope (TS-100) was from Nikon Co. (Tokyo, Japan). The ELISA reader (Mulitiskan) was from Thermo (Fremont, CA, USA). The spectrophotometer (DU 6408) was from Beckman Co. (Fullerton, CA, USA).

A polymeric C30 reversed-phase column (250 × 4.6 mm I.D, particle size 5 μm) from Waters Co. (Milford, MA, USA) was used to separate carotenoids, whereas a Vydac 201TP54 C18 column (250 × 4.6 mm I.D. particle size 5 μm) from Vydac Co. (Hesperia, CA, USA) was used to separate chlorophylls.

### Fractional extraction and preparation of *G. pentaphyllum*

Saponins from *G. pentaphyllum* were extracted and prepared as described previously 4. A method based on Tsai *et al*. 2 was modified to extract and prepare carotenoids and chlorophylls from *G. pentaphyllum*. Briefly, a 10 g powder sample of *G. pentaphyllum* was mixed with 50 ml of hexane-ethanol-acetone-toluene (10:6:7:7, v/v/v/v), after which the mixture was shaken for 1 hr, followed by adding 30 ml of hexane and shaking for 1 min., adding 10% sodium sulphate solution and shaking for 1 min. Then the supernatant was collected, and 30 ml of hexane was added again and extracted repeatedly for four times. All the supernatants were pooled and evaporated to dryness under vacuum, after which the residue was dissolved in 10 ml of hexane to obtain carotenoid and chlorophyll extract for open-column chromatography. Initially a mixture containing 2 g of magnesium oxide and 5 g of diatomaceous earth was poured into a glass column (280 × 15 mm I.D), which was previously activated with 100 ml of hexane, followed by adding anhydrous sodium sulphate to form a layer of around 1 cm above the adsorbent. The 0.5 ml of crude extract was poured into a column, and 20 ml of hexane was added for equilibrium, followed by eluting carotenoids with 30 ml of ethyl acetate-ethanol (98:2, v/v) and chlorophylls with 100 ml of acetone-ethanol (1:1, v/v). Both fractions were collected separately and evaporated to dryness, then the residue was dissolved in 1 ml of methanol-methylene chloride (1:1, v/v) and filtered through a 0.22-μm membrane filter, and 20 μl was injected for HPLC-mass spectrometry (MS) analysis.

A method based on Inbaraj *et al*. 12 was used to separate the various carotenoids in *G. pentaphyllum*. A C30 column and a gradient solvent system of methanol-acetonitrile-water (84:14:2, v/v/v) and methylene chloride (100%) with flow rate at 1 ml/min and detection wavelength at 450 nm were employed to separate 24 carotenoids within 55 min. Similarly, a method developed by Huang *et al*. 13 was used to separate various chlorophylls in *G. pentaphyllum*. A Hypurity C18 column (150 × 4.6 mm I.D, 5 μm particle size) and a gradient mobile phase of acetone, acetonitrile and methanol was used to separate 15 chlorophylls within 35 min. with flow rate at 1 ml/min. and detection at 660 nm. Both carotenoids and chlorophylls were identified based on comparison of retention time, absorption spectra and mass spectra of unknown peaks with reference standards and with those reported in the literature. The cis isomers of carotenoids were also tentatively identified based on spectra characteristics as described in several previous studies 12. In addition, the APCI mode was used to determine mass spectra of carotenoids with MW scanning range 400–1200 m/z, drying gas flow 7 l/min., nebulizer pressure 10 psi, dry gas temperature 330°C, vaporizer temperature 230°C, capillary voltage 2000 V, charging voltage 2000 V, corona current 4 μA and fragmentor voltage 200 V. Likewise, the APCI mode was used to detect mass spectra of chlorophylls with MW scanning range 500–1000 m/z, drying gas flow 5 l/min., dry gas temperature 350°C, capillary voltage 2000 V, charging voltage 2000 V, corona current 4 μA and fragmentor voltage 200 V.

For quantitation, an internal standard β-apo-8′-carotenal was used to quantify carotenoids and Fast Green FCF for chlorophylls. Both standard curves were prepared by using five different concentrations of carotenoid or chlorophyll standards, with each standard solution containing a fixed amount of internal standard. After HPLC-MS analysis, the various carotenoids or chlorophylls were quantified based on the regression equation of each standard curve and a formula as described previously 7,13.

### MTT assay

Human lung carcinoma cell line A549 were cultured in F12-K medium containing 5% FBS and 100 U/ml of penicillin-streptomycin, followed by incubating in a 37°C incubator under 5% CO_2_. The medium was replaced with fresh medium every 2 days to maintain normal cell growth. After growth to the density at around 90% confluence, cells were washed with PBS twice, and then 0.25% trypsin-EDTA was added to react at 37°C for 2 min. Cells were collected by centrifugation at 1200 r.p.m. (RCF = 230 g) for 5 min., and fresh medium was added for resuspension. For MTT assay, 4 × 10^4^ A549 cells were seeded to each well in a 24-well culture plate. After 24 hr incubation with or without PFTα, the medium was replaced with fresh medium or medium containing different doses of the saponin, carotenoid or chlorophyll fractions extracted from *G. pentaphyllum*. After up to 72 hr incubation, 50 μl of MTT reagent (5 mg/ml) was added to each well and incubated at 37°C for 40 min., followed by adding 200 μl of DMSO to react for 15–30 min., collecting 150 μl of DMSO in a 96-well plate, and measuring absorbance at 570 nm with an ELISA reader. The fresh medium without extraction from *G. pentaphyllum* was used as control treatment. The relative cell survival rate was obtained based on the ratio of absorbance of extraction treatment over that of control treatment.

### Cell cycle assay

A549 cells (1 × 10^6^) were seeded to each 10-cm culture plate and incubated for 24 hrs, after which the medium was removed and replaced with fresh medium or medium containing two doses of 30 and 100 μg/ml of saponin fraction. After 12 to 60 hr incubation, cells were washed with PBS and 0.25% trypsin was added to detach cells, followed by flushing cells with PBS. Cell solution was centrifuged at 1200 r.p.m. (RCF = 230 g) for 5 min., after which the supernatant was removed and cells were fixed with precooled 70% ethanol, followed by centrifuging again at 1200 r.p.m. (RCF = 230 g) for 5 min. to remove supernatant. Then PBS was added to wash three times, and PI buffer was added for staining at 37°C for 30 min., after which the cell cycle of A549 cells was analysed by a flow cytometer.

### Annexin-V and PI staining assay

A549 cells (1 × 10^6^) were cultured in a 10-cm plate and incubated for 24 hrs, after which two doses of 30 and 100 μg/ml of saponin fraction were added and incubated for 12–60 hrs, followed by washing with PBS and detaching cells by trypsin followed by neutralization with culture medium. Next, the cells were collected in a 15-ml centrifuged tube, washed with cold PBS twice, centrifuged at 1200 r.p.m. (RCF = 230 g) for 5 min. to remove supernatant, and 0.1 ml of 1× binding buffer containing 0.14 M NaCl, 2.5 mM CaCl_2_ and 0.01 M Hepes/NaOH (pH 7.4) was added to resuspend cells, followed by adding 5 μl of Annexin-V-FITC and 5 μl of 50 ng/ml PI staining agent. After mixed homogeneously and stained at 25°C for 15 min. in the dark, the cells were analysed by a flow cytometer for apoptosis.

### Western blot analysis

A549 cells (1 × 10^6^) were cultured in a 10-cm plate and incubated for 24 hrs, after which the medium was removed and replaced with fresh medium or medium containing two doses of 30 and 100 μg/ml of saponin fraction and incubated for another 36 hrs. Then the medium was removed and washed with PBS twice before detaching cells with a cell scraper. After centrifuging at 1200 r.p.m. (RCF = 230 g) for 5 min., lysis buffer was added to each treatment, and cells were disrupted with a sonicator for protein release, followed by centrifuging again at 14000 r.p.m. (RCF = 20,800 g; 4°C) for 10 min., collecting the supernatant in a centrifuged tube, and storing at −20°C before use. For protein quantitation, seven concentrations of 0, 25, 50, 125, 250, 500 and 750 μg/ml of bovine serum albumin (BSA) were prepared in buffer, after which the Bio-Rad (Hercules, CA, USA) protein assay dye reagent (500 μl) was added to each BSA solution (25 μl) and reacted at 37°C for 30 min., and the absorbance was measured in a cuvette at 562 nm. The standard curve was prepared by plotting concentration against absorbance. The protein samples (25 μl) were analysed in the same way and quantified based on the standard curve of BSA.

For electrophoresis, protein samples were prepared in 1× SDS gel-loading buffer containing 50 mM Tris-HCl (pH 6.8), 0.1% bromophenol blue, 2% SDS, 5% β-mercaptoethanol and 10% glycerol and subjected to protein separation on 10, 12 or 15% SDS-PAGE. Then the separated protein was transferred onto a nitrocellulose membrane in transfer buffer containing 0.04% SDS, 20% methanol, 40 mM glycine and 50 mM Tris base (pH 8.3) at 4°C, which was stained with Ponceau S and washed with deionized water. Next, the nitrocellulose membrane was immersed in a buffer containing 5% skim milk at 25°C for 20 min. to remove background noise, followed by washing with TBST containing 0.05% Tween 20, 150 mM NaCl and 10 mM Tris-HCl (pH 8.0) and adding the primary antibody for conjugation with protein at 25°C for 1 hr. After washing with TBST five times for 5 min. each, the secondary antibody containing HRP was added for protein conjugation at 25°C for 1 hr. Again, TBST was added for washing five times for 5 min. each, and the SuperSignal West Dura Luminol/Enhancer (Thermo Fisher Scientific) was added to produce chemiluminescence, followed by image expression by using the BX 5 × 7 autoradiograph film (Midwest Scientific, Valley Park, MO, USA) for protein signal and intensity measurement.

### shRNA-mediated p53 knockdown

The anti-p53 shRNA construct containing 5′-CCGGCACCATCCACTACAACTACATCTCGAGATGTAGTTGTAGTGGATGGTGTTTTT-3′ (TRCN0000003756) oligonucleotides driven by U6 promoter in pLKO.1 vector was linearized with *ScaI* and transfected into A549 cells by lipofectamine®300 (Life Technologies, Grand Island, NY, USA). Transfected cells were subjected to 2.5 μg/ml puromycin selection and were clonally expanded for p53 knockdown evaluation by western blotting as well as for further experiments.

### Statistical analysis

All the analyses were conducted independently for at least three times in cells of different passages and the data were analysed based on ANOVA and Duncan’s multiple range test to compare means for significant difference (*P* < 0.05) by employing a SPSS software system (IBM, New York, NY, USA).

## Results

### HPLC-MS analysis of saponins, carotenoids and chlorophylls

**Figure 1:**
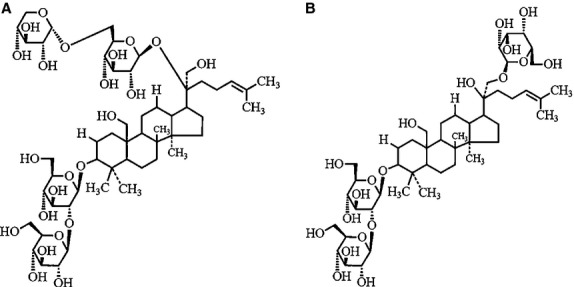
Structures of gypenoside XXII (A) and XXIII (B) in saponin fraction from *Gynostemma pentaphyllum*.

**Figure 2:**
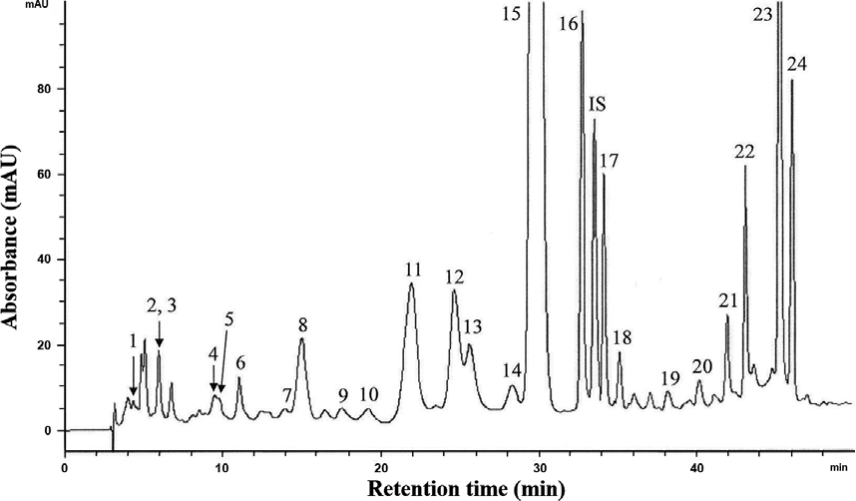
HPLC chromatogram of various carotenoids in carotenoid fraction from *Gynostemma pentaphyllum* (see Table S1 for the values of each integrated peak areas).

**Figure 3:**
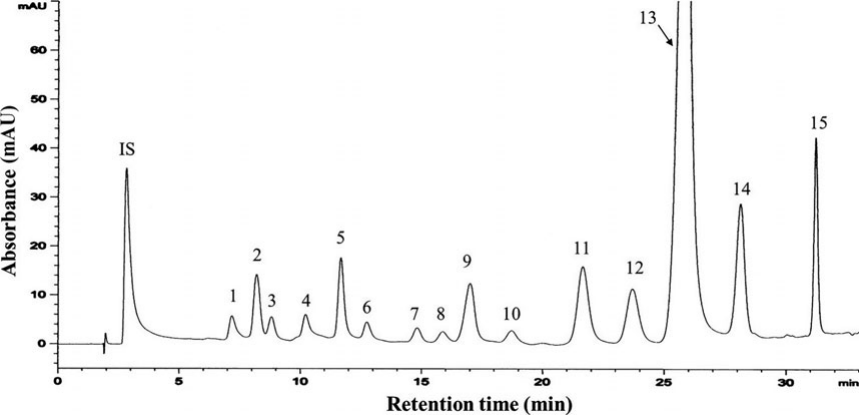
HPLC chromatogram of various chlorophylls in chlorophyll fraction from *Gynostemma pentaphyllum* (see Table S1 for the values of each integrated peak areas).

We have previously developed and reported the extraction and the preparation processes to identify a total of 17 saponins from *G. pentaphyllum* 4. The major components were Gyp XXII and XXIII (Fig.1), representing 43.3% and 27.3% of the total amount in the saponin fraction. All other minor Gyp constituted less than 30%, and any single component accounted for no more than 9% of the fraction as determined by HPLC-MS analysis. By using a similar strategy, carotenoids and chlorophylls from *G. pentaphyllum* were also obtained. Figure2 shows the HPLC chromatogram of various carotenoids in the carotenoid fraction, with a total of 24 carotenoids identified, including auroxanthin (0.5 μg/ml), luteoxanthin (2.3 μg/ml), neoxanthin plus its cis isomers (9.6 μg/ml), lutein plus its cis isomers (129.6 μg/ml), β-cryptoxanthin (1.0 μg/ml), β-carotene plus its cis isomers (67.6 μg/ml) and α-carotene (14.1 μg/ml), with the total amount being 224.7 μg/ml (Table1). Likewise, the HPLC chromatogram of various chlorophylls in the chlorophyll fraction is shown in Figure3, with a total of 15 chlorophylls and their derivatives identified, including hydroxychlorophylls a and b (94.7 μg/ml), chlorophylls a and b (62.4 μg/ml), chlorophylls a′ and b′ (18.1 μg/ml), hydroxypheophytins a and b (70.3 μg/ml), hydroxypheophytins a′ and b′ (196.4 μg/ml), pheophytins a and b (1191.4 μg/ml), pheophytins a′ and b′ (180.3 μg/ml) and pyropheophytin a (50.2 μg/ml), and the total amount was 1866.8 μg/ml (Table2).

**Table 1:** Contents (μg/ml) of various carotenoids in carotenoid fraction from *Gynostemma pentaphyllum*

Peak no.	Carotenoids	Content (μg/ml)*
1	Auroxanthin	0.5 ± 0.1
2	Violaxanthin	–
3	Luteoxanthin	2.3 ± 0.3
4	Neoxanthin	1.2 ± 0.3
5	Cis-neoxanthin	0.9 ± 0.3
6	Cis-neoxanthin	1.8 ± 0.5
7	Cis-neoxanthin	0.7 ± 0.1
8	Cis-neoxanthin	3.8 ± 0.9
9	Cis-neoxanthin	1.2 ± 0.2
10	9 or 9′-cis-lutein	0.9 ± 0.5
11	13 or 13′-cis-lutein	7.6 ± 1.8
12	13 or 13′-cis-lutein	4.6 ± 1.1
13	Cis-lutein	3.2 ± 0.5
14	Cis-lutein	1.4 ± 0.2
15	All-trans-lutein	100.2 ± 10.1
16	9 or 9′-cis-lutein	6.2 ± 1.3
17	Cis-lutein	2.2 ± 0.8
18	9 or 9′-cis-lutein	1.5 ± 0.2
19	Cis-lutein	1.8 ± 0.2
20	All-trans-β-cryptoxanthin	1.0 ± 0.1
21	13 or 13′-cis-β-carotene	2.8 ± 1.2
22	All-trans-α-carotene	14.1 ± 6.5
23	All-trans-β-carotene	48.6 ± 15.8
24	9 or 9′-cis-β-carotene	16.2 ± 5.1
	Total	224.7

* Average of duplicate analyses ± SD.

**Table 2:** Contents (μg/ml) of various chlorophylls in chlorophyll fraction from *Gynostemma pentaphyllum*

Peak no.	Chlorophyll	Content (μg/ml)*
1	Hydroxychlorophyll b	63.5 ± 7.1
2	Chlorophyll b	33.5 ± 5.6
3	Chlorophyll b’	9.3 ± 6.3
4	Hydroxychlorophyll a	31.2 ± 10.3
5	Chlorophyll a	28.9 ± 8.5
6	Chlorophyll a’	8.8 ± 0.5
7	Hydroxypheophytin b	22.7 ± 7.1
8	Hydroxypheophytin b’	53.8 ± 6.9
9	Pheophytin b	141.2 ± 20.2
10	Pheophytin b’	34.9 ± 1.5
11	Hydroxypheophytin a	47.6 ± 1.6
12	Hydroxypheophytin a’	142.6 ± 21.1
13	Pheophytin a	1053.2 ± 50.5
14	Pheophytin a’	145.4 ± 23.8
15	Pyropheophytin a	50.2 ± 8.1
	Total	1866.8

* Average of duplicate analyses ± SD.

### Antiproliferation effect on A549

**Figure 4:**
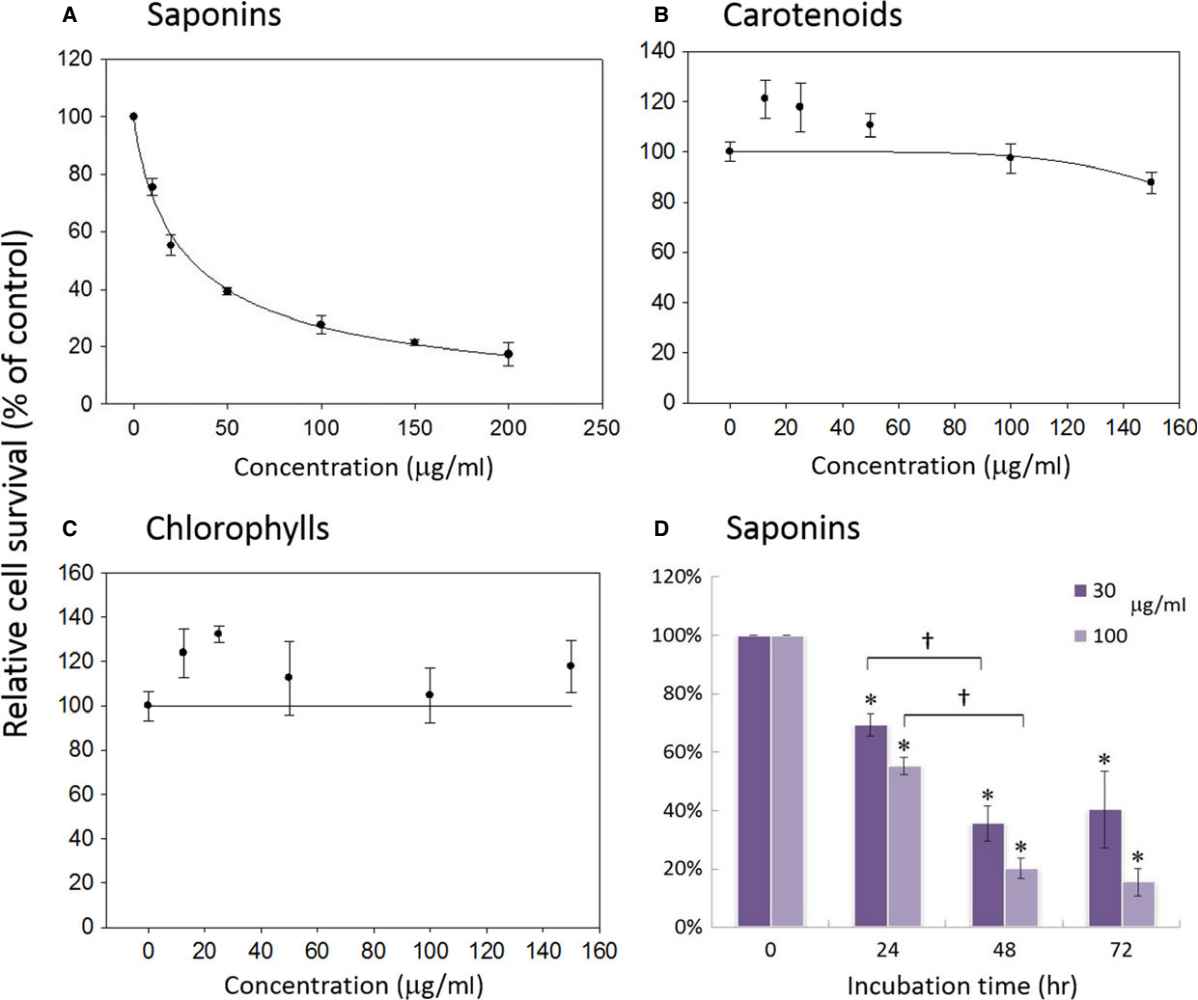
Inhibition effect of saponin (A), carotenoid (B) and chlorophyll (C) fractions from *Gynostemma pentaphyllum* on A549 cell growth as determined by MTT assay after 72 hrs of treatment. Data are shown as averages with indicated standard deviations (*n* = 3). The estimated half maximal inhibitory concentration (IC_50_) of saponin fraction is 30.6 μg/ml. (D) Inhibition effect of saponin fraction on A549 cell as affected by incubation time. Data are shown as averages with indicated standard deviations (*n* = 5). Data with asterisks (*) and crisscross (†) indicate statistic significant (*P* < 0.05) as compared with control and between two groups, respectively.

Figure4 shows the inhibitory effect of the saponin, carotenoid and chlorophyll fractions on A549 cell growth as determined by MTT assay. The saponin fraction showed a concentration-dependent inhibition in A549 cells over the dose range from 10 to 200 μg/ml. With saponin dose of 10 μg/ml, the A549 cell survival rate was 75 ± 3%, followed by a concentration-dependent decline, and an 83 ± 4% inhibition was attained at 200 μg/ml. The estimated half maximal inhibitory concentration (IC_50_) of the saponin fraction was 30.6 μg/ml. In contrast, both carotenoid and chlorophyll fractions did not exhibit effective growth inhibition on A549 up to a dose of 150 μg/ml. The extents of inhibition were further investigated for different incubation periods and the result shown in Figure4D. With saponin fraction treatment at low and high doses of 30 and 100 μg/ml, respectively, a time-dependent growth inhibition of A549 cells was observed over an incubation period of 24–72 hrs. More specifically, 69 ± 3%, 36 ± 6% and 40 ± 13% of A549 cells survived after treatment with 30 μg/ml of saponins for 24, 48 and 72 hrs, respectively. At a concentration of 100 μg/ml, 55 ± 3%, 20 ± 4% and 16 ± 5% of A549 cells survived after 24, 48 and 72 hrs. The maximal growth inhibition of A549 cells could be observed after 48–72 hrs depending on concentrations of the treatment.

### Effects of gypenosides on A549 cell cycles

**Figure 5:**
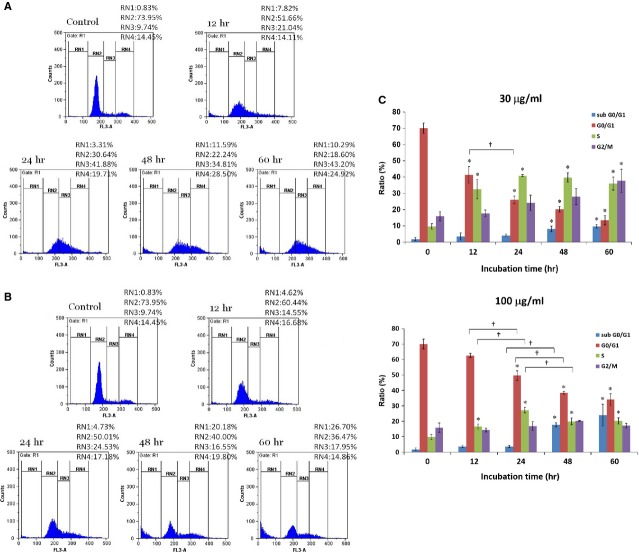
Effects of 30 (A) and 100 (B) μg/ml gypenosides at indicated time intervals on cell cycles of A549 cells analysed by flow cytometry. Cell population percentages of sub-G0/G1, G0/G1, S and G2/M phases are indicated as RN1, RN2, RN3 and RN4%, respectively. Statistical analyses (C) of experimental data shown as averages with indicated standard errors (*n* = 3). Data with asterisks (*) and crisscross (†) indicate statistic significant (*P* < 0.05) as compared with control and between two groups, respectively.

The cell cycles of A549 cells with Gyp were examined and the results are shown in Figure5. Both low (30 μg/ml) and high (100 μg/ml) doses of Gyp could induce aberrant distribution at different phases of the A549 cells. Representative results are shown in Figure5A and B, respectively. A time-dependent reduction of A549 cells at the G0/G1 phase was clearly observed after treating with either low (from 70 ± 3% in controls to 41 ± 5%, 26 ± 2%, 20 ± 2%, and 13 ± 3% after 12, 24, 48 and 60 hrs, respectively) or high (from 70 ± 3% in controls to 63 ± 1%, 50 ± 3%, 38 ± 1%, and 34 ± 4% after 12, 24, 48 and 60 hrs, respectively) concentration of Gyp (Fig.5C). It is interesting to note that the reduction was more prominent when treating with 30 μg/ml than that with 100 μg/ml of Gyp.

In contrast, an increase of A549 cells at both the S phase and the G2/M phase were observed following Gyp treatment. As summarized in Figure5C, the A549 cells at the S phase increased from 10 ± 2% in controls to 33 ± 6%, 41 ± 1%, 40 ± 3% and 36 ± 4% after treating with low concentration of Gyp for 12, 24, 48 and 60 hrs, respectively. At high concentration, the increase was only apparent after 24 hrs of treatment, but to a much less extent, from 10 ± 2% in controls to 17 ± 2%, 27 ± 2%, 20 ± 2% and 20 ± 2% after 12, 24, 48 and 60 hrs of treatment, respectively. A plateau of 41% and 27% cells at the S phase was attained after 24 hrs with both low and high doses of Gyp treatment. A time-dependent increase of A549 cells at the G2/M phase was also observed when treating with low (from 16 ± 3% in controls to 18 ± 2%, 24 ± 5%, 28 ± 5% and 38 ± 7% after 12, 24, 48 and 60 hrs, respectively), but not high (from 16 ± 3% in controls to 14 ± 1%, 17 ± 3%, 20 ± 1% and 17 ± 2% after 12, 24, 48 and 60 hrs, respectively) doses of Gyp.

Similarly, a time-dependent increase of A549 cells at the sub-G0/G1 phase, representing the apoptotic or necrotic population, did occur among A549 after Gyp treatment. With a dose of 30 μg/ml, more A549 cells were found at the sub-G0/G1 phase after Gyp treatment (from 2 ± 1% in controls to 4 ± 2%, 4 ± 1%, 8 ± 2%, and 10 ± 1% after 12, 24, 48 and 60 hrs, respectively). The differences became more pronounced (from 2 ± 1% in controls to 4 ± 2%, 4 ± 1%, 18 ± 1%, and 24 ± 7% after 12, 24, 48 and 60 hrs, respectively) when the dose was raised to 100 μg/ml and the incubation time was prolonged.

### Apoptotic effects of gypenosides on A549 cells

**Figure 6:**
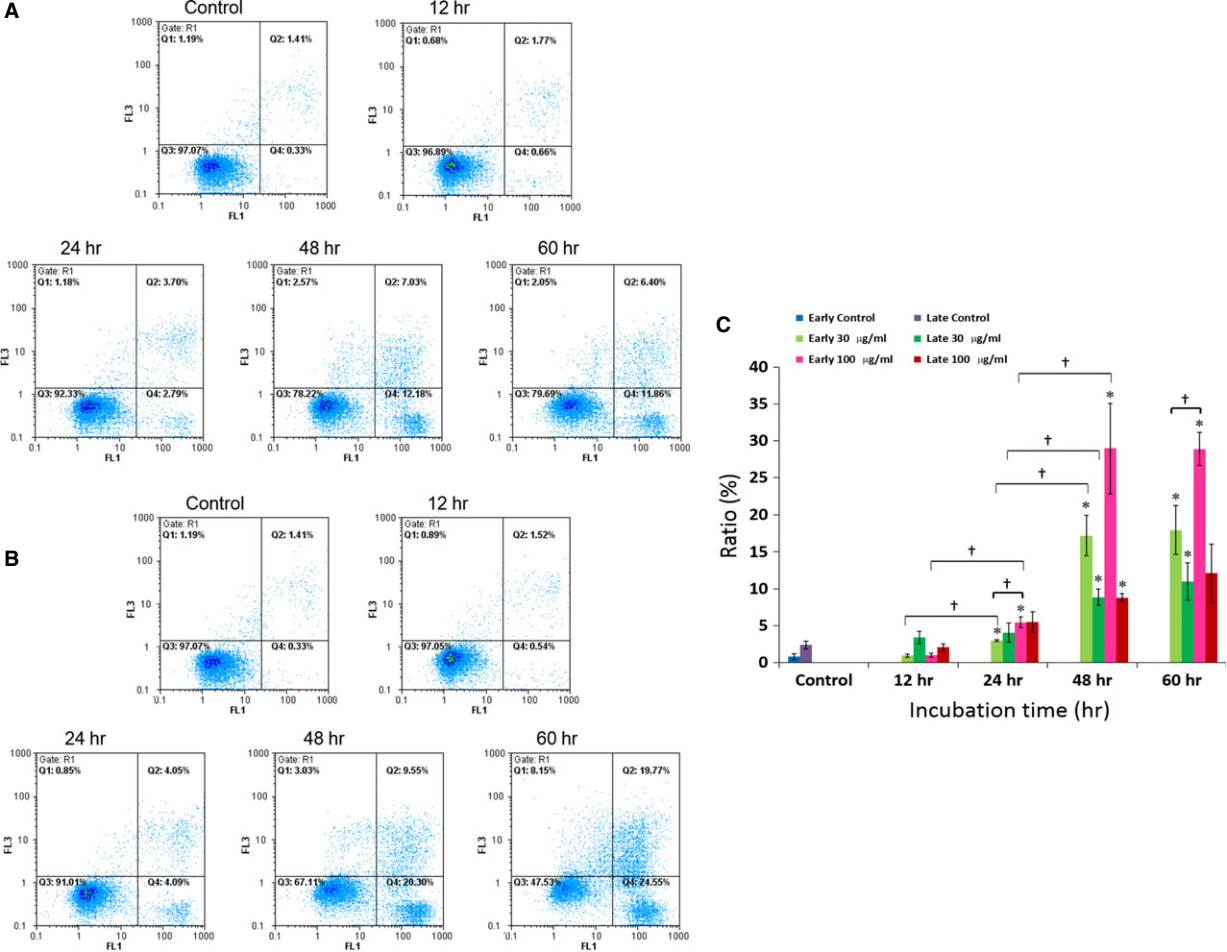
Annexin-V analysis by a flow cytometry of A549 cells treated with 30 (A) and 100 (B) μg/ml gypenosides from *Gynostemma pentaphyllum*. Q3, normal; Q4, Annexin-V positive (*i.e*. early apoptotic population); Q1, PI positive; Q2, Annexin-V/PI positive (*i.e*. late apoptotic population). Statistical analyses (C) of experimental data shown as averages with indicated standard errors (*n* = 3). Data with asterisks (*) and crisscross (†) indicate statistic significant (*P* < 0.05) as compared with control and between two groups, respectively.

The Annexin-V assay is commonly used to determine the apoptotic cell population based on flow cytometry. Accordingly, during cell apoptosis, phosphatidyl serine translocates from the inner plasma membrane leaflet to the outer one for conjugation with Annexin-V to show the Annexin-V positive phenomenon in the area Q4, which is an indication of the early stage of apoptosis. In contrast, during the late stage of apoptosis, the breakdown of plasma membrane causes direct conjugation between PI and DNA to show the Annexin-V/PI positive phenomenon in the area Q2 due to the addition of both Annexin-V and PI at the same time. Figure6 clearly shows that a time-dependent rise in the proportions of A549 cells underwent both early and late stages of apoptosis after low (30 μg/ml) and high (100 μg/ml) doses of Gyp treatment. As summarized in Figure6C, the early and late apoptotic A549 cells increased from 0.8 ± 0.4% in controls to 0.9 ± 0.2%, 3.0 ± 0.1%, 17.2 ± 2.7% and 18.0 ± 3.3%, as well as from 2.3 ± 0.5% in controls to 3.4 ± 0.8%, 4.0 ± 1.3%, 8.9 ± 1.1% and 11.0 ± 2.6% after 12, 24, 48 and 36 hrs of treatment with low dose of Gyp, respectively. More prominently, the early and late apoptotic A549 cells increased from 0.8 ± 0.4% in controls to 1.0 ± 0.3%, 5.5 ± 0.1%, 29.0 ± 6.1% and 28.9 ± 2.3%, as well as from 2.3 ± 0.5% in controls to 2.1 ± 0.5%, 5.5 ± 1.4%, 8.8 ± 0.6% and 12.1 ± 4.0% after 12, 24, 48 and 36 hrs of treatment at high dose. A plateau of 18% and 29% of the early apoptotic A549 cells was attained after 48 hrs of Gyp treatment at both low and high doses, respectively.

### Expression of proteins associated with cell cycle control and apoptosis

**Figure 7:**
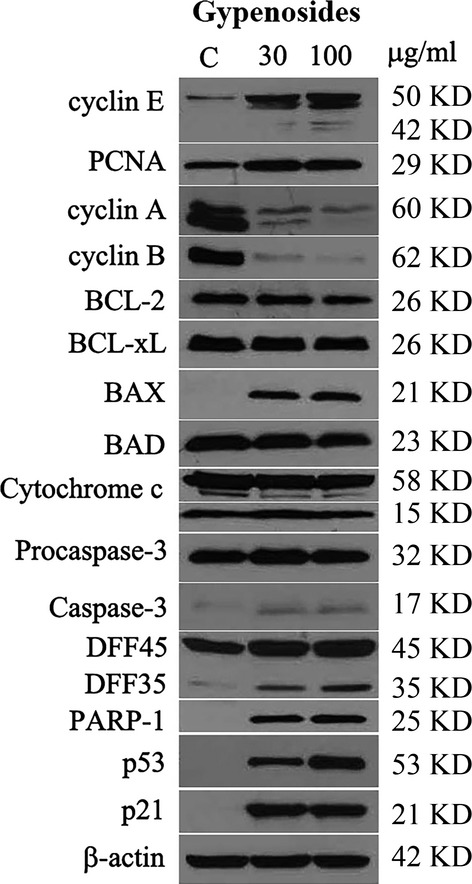
Expression of selected cellular proteins in A549 cells treated with gypenosides from *Gynostemma pentaphyllum*. β-actin was used as a protein loading control.

The expression profiles of selected proteins associated with cell cycle control and apoptosis in A549 cells as affected by Gyp are shown in Figure7. As described earlier, Gyp treatment could induce an arrest at both S phase and G2/M phase, as well as apoptosis in the A549 cells. Thus, the expression of cell cycle regulatory proteins cyclins, and anti-apoptotic proteins such as BCL family, caspases and downstream apoptotic effectors in A549 cells treated with Gyp was further investigated using β-actin as a protein loading control. An increased expression in cyclin E (∽six- and nine-fold of that in control, respectively, as determined by a densitometer) and proliferating cell nuclear antigen (PCNA; ∽1.5-fold of that in control at both doses) was observed in A549 cells treated with both 30 and 100 μg/ml of Gyp. In contrast, a concentration-dependent decline in the expression of cyclin A and B was shown in A549 cells, with 0.3- and 0.2-fold (cyclin A) as well as 0.1- and 0.05-fold (cyclin B) of that in control after Gyp treatment at doses of 30 and 100 μg/ml, respectively. These results indicate that Gyp form *G. pentaphyllum* indeed modulate the expression of various regulatory proteins to affect the cell cycle control in the A549 cells.

The expression of BCL-2 also followed a decreasing trend, which was 0.9- and 0.7-fold of that in control after treatment with 30 and 100 μg/ml Gyp, respectively. But an obvious increase in protein amount was clearly detected for the BAX following both 30 and 100 μg/ml of Gyp treatment (the log2 values of relative protein expression are shown in Fig. S1). As for BCL-xL, BAD, cytochrome c and procaspase-3, no obvious change in protein expression was noted after treatment with Gyp. However, small but elevated amount of activated caspase-3 was clearly detected after Gyp treatment. Activation of caspase-3 by Gyp also led to activation of the downstream effectors, such as the cleavage of DFF45 and an increased expression of PARP-1, further supporting that apoptosis did occur in A549 cells treated with Gyp. In addition, the expression of the p53 and p21 was also significantly induced after treatment with Gyp at both doses of 30 and 100 μg/ml. Taken together, above results suggest that treatment of Gyp disturbed the balance on the expressions of anti-apoptotic protein BCL-2 and pro-apoptotic protein BAX, leading to activation of caspase-3 to execute apoptosis in the A549 cells.

### The role of p53 in gypenoside induced A549 growth inhibition

**Figure 8:**
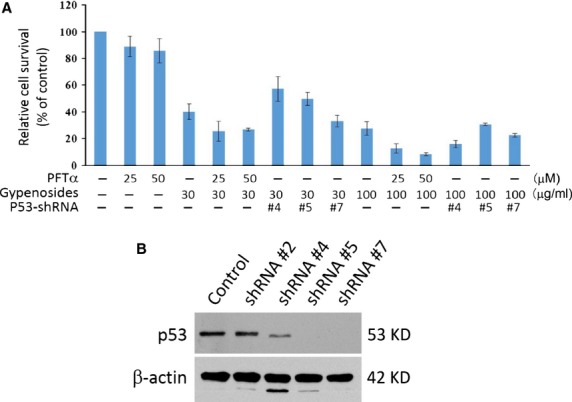
Effect of p53 on gypenoside-induced A549 growth inhibition. (A) A549 cell survival in the presence or absence of gypenosides (30 or 100 μg/ml), pifithrin-α (PFTα, a p53 inhibitor, 25 or 50 μM) and p53-shRNA (clone #4, 5 and 7). (B) Cellular p53 protein levels in four independent A549 clones stably transfected with anti-p53-shRNA.

Both a p53 inhibitor, PFTα, and the shRNA-mediated p53 knockdown in A549 cells were utilized to evaluate the role of p53 in Gyp-induced growth inhibition. As shown in Figure8A, the treatment of PFTα in A549 cells slightly inhibited the growth by 11 ± 7 and 14 ± 9% at concentrations of 25 and 50 μM, respectively. However, the addition of PFTα at both concentrations did not abolish A549 growth inhibition induced by Gyp treatment. At the dose of 30 μg/ml Gyp, the A549 cell survival rate was 40 ± 6%, which was reduced to 26 ± 7 and 27 ± 1% in the presence of 25 and 50 μM of PFTα, respectively. At 100 μg/ml Gyp, the cell survival rate was 27 ± 5%, which was 13 ± 3 and 8 ± 1% in the presence of 25 and 50 μM of PFTα, respectively. Similarly, three independent clones of A549 cells stably transfected with shRNA to mediate p53 knockdown also retained similar sensitivity to Gyp-induced growth inhibition. The cell survival rate ranged from 33–57% and 16–31% in the presence of 30 and 100 μg/ml of Gyp, respectively, which were comparable to those in A549 cells without shRNA-mediated p53 knockdown. Decreased cellular expression of p53 protein in these independent A549 cell clones were demonstrated in Figure8B. An apparent reduction of cellular p53 was observed in clone #4, whereas no detectable p53 protein was found in both clone #5 and 7. β-actin was included as a protein loading control.

## Discussion

Lung cancer is becoming the leading cause of cancer deaths around the world, so it is imperative to find a possible adjuvant treatment for this disease. Previous studies have used *G. pentaphyllum* extracts as raw materials to access their antiproliferative effects on cancer cells or as possible treatments for chronic diseases 1,14,15. However, many of the details of the molecular mechanisms underlying those bioactive properties remain unclear. In this study, we used open-column chromatography to prepare and purify saponins, carotenoids and chlorophylls from *G. pentaphyllum* extract in order to investigate their inhibitory effects on lung carcinoma A549 cells, as well as to explore the potential cellular mechanisms involved, which should provide direct evidence for the beneficial impacts of the relevant functional components on human health.

According to MTT assay results, the saponin fraction was superior to both the carotenoid and chlorophyll fractions in suppressing the growth of lung carcinoma A549 cells. This is consistent with our previous report that saponins from *G. pentaphyllum* exhibit antiproliferative effects on the PC-3 prostate cancer cell line 4, in addition to other reports on various cancer cell lines 3,5,6. A similar growth inhibitory effect on A549 cells induced by Gyp, triterpenoid saponin glycosides extracted from *G. pentaphyllum*, has also been reported by Lu *et al*. 11. However, the estimated IC_50_ of their Gyp preparation (∽110 μg/ml) was at least threefold higher than that of ours (30.6 μg/ml), indicating that the active component(s) against A549 cell growth was(were) more abundant and/or better synergized in our preparation. However, in contrast with a recent report from Tsai *et al*. indicating that carotenoids and chlorophylls from *G. pentaphyllum* showed antiproliferative effects on hepatoma Hep3B cells, no significant growth inhibition on A549 cells was observed for those fractions in the present study. As shown in Table1, lutein and its *cis* isomers made up the largest portion (129.6 μg/ml) of the carotenoid fraction, while pheophytin and its derivatives (1691.6 μg/ml) were the main components of the chlorophyll fraction. These compounds extracted from *G. pentaphyllum* may not be effective for the antiproliferation of A549 cells. Nonetheless, antiproliferative effects for lutein and several other carotenoids, such as neoxanthin and β-carotene, have been reported for prostate cancer, oesophageal cancer and lymphoid leukaemia cell lines 16,17. In addition, the antiproliferative effects of pheophytin a and b have been reported for liver cancer cell lines 18. These contrasting observations imply that the inhibition efficacy of carotenoids and chlorophylls on cancer cells may depend on the specific carotenoids, chlorophylls and cancer cell types in question.

As the saponin fraction, which consisted mainly of Gyp XXII and XXIII, exhibited a better growth inhibition on A549 cells, further investigations were focused on the effect of this Gyp containing fraction at concentrations of 30 (IC_50_) and 100 μg/ml (which inhibited A549 growth by more than 75% after 72 hrs). Our data demonstrated an apparent arrest of A549 growth at both the S phase and G2/M phase following treatment with 30 μg/ml Gyp for 12 hrs and this arrest persisted for up to 60 hrs. This finding was further supported by western blotting assay results, which indicated that the incubation of A549 with Gyp resulted in a prominent decline of cellular expression of cyclin A and B in a concentration-dependent manner, as well as an increase in the expression of PCNA, a marker of the S phase. Both cyclin A and B are important for the G2-M transition. Cyclin A is the only cyclin that regulates multiple steps of the cell cycle *via* its ability to associate with two distinct cyclin-dependent kinases, CDK1 and 2 19,20. This association of cyclin A with CDK2 and CDK1 is required for cellular passage into the S phase and for entry into the M phase, respectively 21, whereas the association of cyclin B with CDK1 regulates the progression from the G2 to the M phase 22. Therefore, expression modulations of PCNA as well as cyclin A and B are consistent with the observed elevation of the A549 population at the S phase and with the impact on the G2-M transition in A549 cells after Gyp treatment, respectively.

In contrast, Lu *et al*. previously reported that Gyp (200–400 μg/ml) induced a partial arrest of the A549 cell cycle at the G0/G1 phase, even though a transient increase in the A549 population at the G2/M phase following incubation with 150 μg/ml of Gyp (IC_50_ 110 μg/ml) and an apparent increase in the sub-G0/G1 population following 300 μg/ml of Gyp treatment for 48 hrs were also observed 11. These discrepancies with regard to observations of A549 cell cycle arrest induced by Gyp may possibly be attributed to the differences in the composition of Gyp obtained *via* different preparation methodologies and/or from *G. pentaphyllum* supplied by different sources. Unlike Lu *et al*., who primarily enriched Gyp A, C and E from *G. pentaphyllum* 11, those components only accounted for 8.9% of our preparation content 4. Instead, Gyp XXII and XXIII were the major components of our saponin fraction, constituting 43.3% and 27.3%, respectively. These observations strongly suggest that discrete forms of bioactivity, specifically in terms of effects on the cell cycle of A549, may indeed exist among different glycosylation varieties of dammarane triterpene and/or be complicated by synergistic effects in combination. In addition, our western blotting results indicated that the expression of cyclin E was also increased in a concentration-dependent manner, suggesting that possible modulation of the G1-S transition of A549 cells may also be in effect, although to a lesser extent. Cyclin E/CDK2 promotes the progression from the G1 to the S phase by phosphorylating retinoblastoma protein (Rb) to release the E2F transcriptional factor that drives the expression of genes for this transition 23. Over-expression of cyclin E has been shown to be involved in various types of cancers, including breast, colon, bladder, skin and lung cancer, findings which indicate its role in tumourigenesis 24. In combination, dysregulation of cyclin A, B and E after saponin treatment may result in progression through the G1-S transition followed by a defective DNA replication and a block in the G2-M transition, effects which would lead to the observed S and G2/M arrest in A549. Furthermore, as the treatment of Gyp was prolonged and/or a high dose (100 μg/ml) was used, a more significantly increased percentage of the A549 population at the sub-G0/G1 phase was observed. These results suggested that although Gyp at a concentration around IC_50_ (30.6 μg/ml) is sufficient to cause S and G2/M arrest in A549 cells, higher concentrations and prolonged treatment with Gyp may lead to cellular destruction, such as apoptosis. A similar observation was made in our prior report on prostate cancer PC-3 cells following Gyp treatment 4. A significant increase in the A549 population at the sub-G0/G1 phase following Gyp treatment was also reported by Lu *et al*., strongly suggesting that the occurrence of A549 cell death after Gyp treatments.

Apoptosis is a form of programmed cell death with characteristic morphological features resulting from the activation of caspase family of cellular proteases. Our results also demonstrated that Gyp do significantly enhance both the early and late apoptotic populations of A549 cells at concentrations of both 30 and 100 μg/ml after 48 hrs. The expression modulation of apoptosis-mediating molecules is commonly observed in cancer cells following treatment with antitumour compounds. For example, BCL-2 and BAX, which function as an intracellular suppressor and stimulator of apoptosis, respectively, have been shown to be down- and up-regulated, respectively, in the A549 cells after treatment with Gyp 11. Accordingly, both pro-apoptotic proteins BAX and BAD as well as anti-apoptotic proteins BCL-2 and BCL-xL should be balanced under normal physiological conditions, but the disruption of their balance can induce cell death or survival as a result of changes in cellular signals. Our results revealed that the house-keeping BCL-2 family proteins in A549 cells, including BCL-2, BCL-xL and BAD, maintain the normal survival of cells. After Gyp treatment, we observed a mitigation of cellular BCL-2 and an obvious induction of BAX expression in the A549 cells, shifting the balance towards pro-apoptotic status and activating the conversion of inactive procaspase-3 to active capase-3. This activation of caspase-3 also resulted in the activation of downstream effectors, such as the proteolysis of DFF45 to DFF35 25 and the induction of PARP-1 26, leading to the apoptosis of the A549 cells. These results further supported the ability of Gyp to induce A549 apoptosis. However, Lu *et al*. observed expression modulation of BAX and BCL-2 in A549 cells treated with Gyp at a concentration of 300 μg/ml. We observed a similar effect at concentrations of 30 and 100 μg/ml. Since Gyp A, C and E were enriched by Lu *et al*. 11, whereas Gyp XXII and XXIII were the major components of our saponin preparation, the above results strongly imply that Gyp XXII and XXIII may be more potent than Gyp A, C and E in inducing both cell cycle arrest and apoptosis in A549 cells.

The p53 protein, a well-known tumour suppressor, mediates pathways involved in both cell cycle arrest and apoptosis 27. Once activated, it functions as a transcriptional factor to enhance the expression of the downstream protein p21, which inhibits several cyclin/CDK complexes and therefore prevents the cell cycle from progressing. In addition, the transcriptions of both BAX and cyclin A have been shown to be regulated and modulated, respectively, by the p53 protein 20,28. Cellular levels of cyclin B have also been reported to be inversely regulated by the p53 protein 29. The binding of p21 to the carboxy-terminal domain of PCNA was previously demonstrated to abolish its ability as a processivity factor for DNA polymerase 30. Our data showed that an apparent concentration-dependent induction of the p53 protein in A549 cells occurred following Gyp treatment. This induction of the p53 protein in A549 also resulted in expression induction of the p21 protein, suggesting that cell cycle arrest and apoptosis of A549 cells induced by Gyp may proceed through a p53-p21 pathway. However, the addition of a p53 inhibitor, PFTα, did not alleviate the A549 growth inhibition induced by Gyp. This observation was further confirmed by the similar growth inhibition effect of saponins on A549 cells with or without shRNA-mediated p53 knockdown. Both results suggest a minimal role of p53 in Gyp-induced A549 growth inhibition; thus, the cell cycle arrest and apoptosis of A549 cells induced by Gyp would most likely proceed regardless of the cellular status of p53, *i.e*. through p53-independent pathway(s).

In addition to mediating p53-dependent G1 arrest, the p21 protein has been reported to inhibit progression through the S phase to the G2 phase and the G2/M transition by suppressing the activities of the CDK1/2-cyclin A and CDK1-cyclin B1 complexes, respectively, in a p53-independent manner 31. In addition, various types of DNA damage, hypoxia, aberrant cellular ribonucleoside triphosphate pools and other stressful signals have been reported to induce p53 expression 27. Further investigation will be needed to determine the upstream events that initiate the induction of p53 expression as well as the genuine signal pathway(s) that initiate A549 cell cycle arrest and apoptosis following Gyp treatment. The p53 protein has long been an attractive target for cancer therapy development due to its nearly universal inactivation either by mutations or by accelerated cellular degradation in human malignancies 32. However, the induction of p53-independent growth inhibition in lung carcinoma A549 cells by Gyp suggests that the utilization of Gyp has potential as an adjuvant treatment for this disease.

## Conclusion

In the present study, the saponins, carotenoids, and chlorophylls from *G. pentaphyllum* were isolated and evaluated for their antiproliferative effects on lung carcinoma A549 cells. The saponin fraction, which consisted mainly of Gyp XXII and XXIII, form *G. pentaphyllum* was superior to both the carotenoid and chlorophyll ones in suppressing the growth of the A549 cells with a concomitant induction of A549 cell cycle arrest at both the S phase and the G2/M phase. A concentration-dependent decrease in the expression of the cell cycle regulatory proteins, cyclin A and B, as well as an increase in cyclin E and PCNA expression were detected, which are in concordance with the observed S and G2/M arrest in A549 cells after Gyp treatment. In addition, a concentration-dependent decrease in the expression of the anti-apoptotic proteins BCL-2 and an increase in the expression of the pro-apoptotic proteins BAX, as well as the activation of caspase-3 and the downstream substrates, DFF45 and PARP-1, were shown, indicating the induction of apoptosis in A549 cells by Gyp. Although the Gyp-induced expression of the p53 and the downstream p21 proteins was observed in A549 cells, both treatment of a p53 inhibitor, pifithrin-α (PFTα), and the shRNA-mediated p53 knockdown in A549 cells did not apparently abolish the growth inhibition effect induced by Gyp. All these findings suggest that the Gyp-induced cell cycle arrest and apoptosis in A549 cells may proceed through p53-independent pathway(s).

## Supporting Information

**Figure S1** Expression quantitation based on densitometry of western blotting data of selected cellular proteins in A549 after Gyp treatment. **Table S1** The average values of each peak areas in HPLC chromatograms of the carotenoid (A) and chlorophyll (B) fractions.
