# Supplementary material for: Induction of p53-independent growth inhibition in lung carcinoma cell A549 by gypenosides
Source: J Cell Mol Med. 2015 Mar 17;19(7):1697–709. doi: 10.1111/jcmm.12546 (PMC4511366; doi:10.1111/jcmm.12546)
Supplement: Supplementary file 1 [file jcmm0019-1697-sd1.docx]

**Supplementary Information**

**Table S1.** The average values of each peak areas in HPLC chromatograms of the carotenoid (A) and chlorophyll (B) fractions.

1. **(B)**


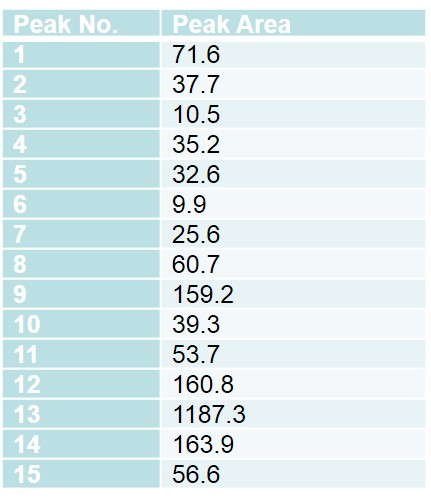

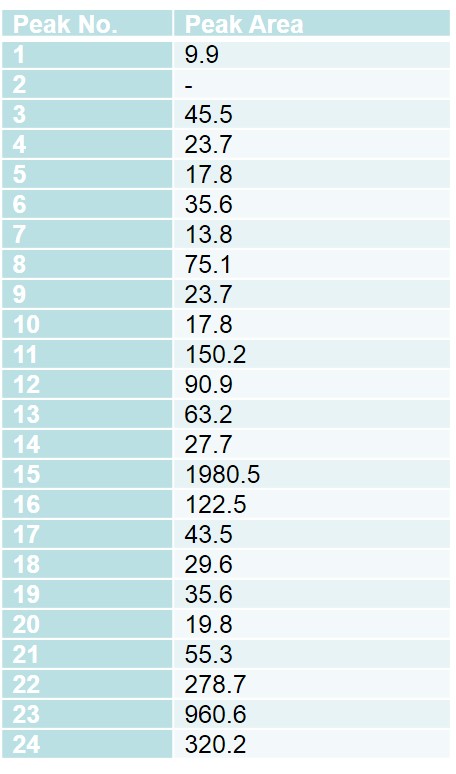


**Figure S1.** Expression quantitation based on densitometry of western blotting data of selected cellular proteins in A549 after Gyp treatment.
